# Five-Year Follow-Up of Parapapillary Atrophy: The Beijing Eye Study

**DOI:** 10.1371/journal.pone.0032005

**Published:** 2012-05-07

**Authors:** Yin Guo, Ya Xing Wang, Liang Xu, Jost B. Jonas

**Affiliations:** 1 Beijing Institute of Ophthalmology, Beijing Tongren Hospital, Capital Medical University, Beijing, China; 2 Department of Ophthalmology, Medical Faculty Mannheim of the Ruprecht-Karls-University Heidelberg, Germany; Massachusetts Eye & Ear Infirmary - Harvard Medical School, United States of America

## Abstract

**Purpose:**

To assess longitudinal changes in parapapillary atrophy in the adult population of Greater Beijing.

**Methods:**

The population-based Beijing Eye Study 2006 included 3251 subjects who had participated in the Beijing Eye Study 2001 and returned for re-examination. The mean age was 60.4±10.1 years. Using optic disc photographs, we measured parapapillary atrophy which was divided into alpha zone and beta zone.

**Results:**

Overall progression rate of alpha zone was seen in 0.6±0.1% (95% confidence interval (CI):0.3,0.9) of the subjects and of beta zone in 8.2±0.5% (95%CI:7.2,9.1) of the subjects. In binary regression analysis, rate of progression of alpha zone was significantly associated higher age (*P* = 0.04) and the co-progression of zone Beta (*P*<0.001). Rate of progression of beta zone was significantly associated with higher age (*P*<0.001; odds ratio (OR):1.11;95%CI:1.10,1.14), higher intraocular pressure (*P*<0.001;OR:1.10;95%CI:1.05,1.14), higher myopic refractive error (*P*<0.001;OR:0.71; 95%CI:0.67,0.75), rural region of habitation (*P* = 0.002;OR: 0.58; 95%CI:0.41,0.82), presence of glaucomatous optic nerve damage (*P*<0.001;OR:2.89; 95%CI:1.62,5.14), co-progression of alpha zone (*P*<0.001;OR:7.13;95%CI:2.43,20.9), absence of arterial hypertension (*P* = 0.03;OR: 0.70; 95%CI:0.51,0.96), and thicker central corneal thickness (*P* = 0.02;OR:1.01;95%CI:1.00,1.01). Subjects with a non-glaucomatous optic nerve damage (n = 22) as compared to the remaining subjects did not vary in the progression rate of alpha zone (0.0% versus 0.6±0.1%; *P* = 1.0) and beta zone (8.2±0.5% versus 6.3±0.6%;*P* = 1.0).

**Conclusions:**

In adult Chinese in Greater Beijing, the 5-year progression rate of beta zone of parapapillary atrophy (seen in 8.2±0.5% of subjects) was significantly correlated with higher age, rural region of habitation, absence of arterial hypertension, higher intraocular pressure, higher myopic refractive error, thicker central corneal thickness, and presence of glaucoma. It was not associated with non-glaucomatous optic nerve damage.

## Introduction

Since the early 20th century, parapapillary atrophy has been recognized as an essential part in the description of the morphology of the optic nerve head in normal eyes, eyes with glaucomatous optic neuropathy, and eyes with a non-glaucomatous optic nerve damage [1]–[5]. Frequency, size, and regional distribution of parapapillary atrophy in Caucasians have been previously examined in several hospital-based studies [6]–[21], and in a cross-sectional study design in the population-based Rotterdam study [22]. For non-white populations, the Beijing Eye Study assessed parapapillary atrophy in Chinese in 2001 [23], [24]. Since glaucoma is associated with an enlargement and increased frequency of beta zone of parapapillary atrophy, and since a change in the appearance of beta zone can be taken to detect a progression of glaucoma, it is important to know the spontaneous rate of change in parapapillary atrophy and the factors associated with such a change. We therefore conducted this study to assess age-related changes in the size and presence of parapapillary atrophy in a population-based study design.

## Methods

### Ethics Statement

The Medical Ethics Committee of the Beijing Tongren Hospital approved the study protocol and all participants gave written informed consent.

The population-based Beijing Eye Study was first performed in the year 2001, and the follow up study was carried out 5 years later in 2006 [24], [25]. At baseline, 4439 subjects out of 5324 eligible subjects participated (response rate: 83.4%). The study was divided into a rural part (1973 subjects; 1143 women) and an urban part (2466 subjects; 1362 women). The mean age was 56.2±10.6 years (median: 56 years; range: 40–101 years). At baseline and at the follow-up examination, a comprehensive eye examination was carried out including visual acuity assessment, perimetry, noncontact tonometry (CT-60 computed tonometer, Topcon Ltd., Tokyo, Japan), slit-lamp examination of the external eye and anterior segment, and photography of the lens (Neitz CT-R camera, Neitz Instruments Co., Tokyo, Japan) and macula and optic disc (fundus camera Type CR6-45NM, Canon Inc. U.S.A.). The visual field examinations were performed by frequency-doubling perimetry using the screening program C-20-1 (Zeiss-Humphrey, Dublin, California, USA). After qualitative assessment, the optic disc photographs were digitized and the optic disc structures were measured by outlining the borders of the optic disc, optic cup, peripapillary scleral ring, and alpha zone and beta zone of parapapillary atrophy border on the computer screen [26], [27]. Parapapillary chorioretinal atrophy was divided into a peripheral alpha zone and a central beta zone at the optic disc border. Alpha zone was characterized by irregular hypopigmentation and hyperpigmentation. Features of beta zone were marked atrophy of the retinal pigment epithelium and of the choriocapillaris, good visibility of the large choroidal vessels and the sclera, thinning of the chorioretinal tissues, and round bound to the adjacent alpha zone on its peripheral side and to the peripapillary scleral ring on its central side. Glaucoma was defined according to the criteria of the International Society of Geographic and Epidemiological Ophthalmology ISGEO [28]. In that definition, criteria for a category 1 diagnosis (structural and functional evidence) were a vertical cup/disc diameter ratio (VCDR) or an inter-eye asymmetry in the VCDR of ≥97.5th percentile for the normal population, or a neuroretinal rim width reduced to ≤0.1 VCDR (between 11 to 1 o'clock or 5 to 7 o'clock), in addition to a definite visual field defect consistent with glaucoma. Criteria for the category 2 diagnosis (advanced structural damage with unproven visual field loss) were a VCDR or a VCDR asymmetry ≥99.5th percentile for the normal population. Criteria for a category 3 diagnosis (for eyes the optic nerve head of which could not be examined or for which a visual field examination was not possible) were a visual acuity <3/60 combined with either an intraocular pressure >99.5th percentile, or definite glaucoma medical records such as filtering surgery history [27]. In this study, a patient was defined as having glaucoma if the criteria for any one of these three categories were met.

In 2006, the study was repeated by inviting all participants of the survey from 2001 to be re-examined. We additionally measured body weight and height and arterial blood pressure, and we took fasting blood samples for biochemical analysis. The concentrations of glucose, cholesterol, low-density lipoproteins and high-density lipoproteins were measured [25]. Death and the corresponding cause were investigated and confirmed by the certificate provided by the local hospital. Axial length was measured for 704 subjects by a partial coherence interferometry analyzer (Lenstar Biometry; LS900, Haag-Streit, Berne, Switzerland).

The optic disc photographs taken in 2001 and in 2006 from the same eye were projected in a random order and in a masked manner. Only the right eyes were examined and included into the study. The photographs of all eligible eyes were examined, independently whether there was alpha zone or beta zone present or not at the baseline examination in 2001. If by comparing both photographs, which were projected for the same eye in a random masked manner, a difference in alpha zone or beta zone of parapapillary atrophy was detected, and if, after unmasking, the larger zone or the new zone was present without doubt on the photograph taken in 2006, the eye was considered to have progressive parapapillary atrophy, defined as enlargement of a zone already existing at baseline or as new development of a zone of parapapillary atrophy. The primary assessment of all photographs was carried out by a trained ophthalmologist (YG) and intermittently controlled by a panel of specialists (YXW, LX, JBJ).

Inclusion criteria for the present study were the availability of assessable optic disc photographs taken in 2001 and 2006. Statistical analysis was performed using a commercially available statistical software package (SPSS for Windows, version 19.0, IBM-SPSS Inc. Chicago, IL). Only one eye per subject (the right eye) was taken for statistical analysis. The frequency of a progression in parapapillary atrophy was given as mean ± standard error. The mean values of all other parameters were given as mean ± standard deviation. The Gaussian distribution of the parameters was tested using the Kolmogorov-Smirnov test. In a first step, we examined the mean values in the group with progression of parapapillary atrophy versus group with stable parapapillary atrophy and we calculated the significance of the differences between both groups using the student-t-test for non-paired samples for the parametric parameters, the Mann-Whitney-U test for non-paired samples for the non-parametric parameters, and the Chi-squared test for the comparison of frequencies (Table 1). In a second step, we performed a binary logistic regression analysis with the progression (versus non-progression) as dependent parameter and those parameters as independent parameters which were significantly associated with progression of parapapillary atrophy in the univariate analysis (Table 2). Odds ratios (OR) were calculated and 95% confidence intervals (CI) were presented. All p-values were 2-sided and were considered statistically significant when the values were less than 0.05.

**Table 1:** Associations of the 5-Year Incidence of Alpha Zone and Beta Zone of Parapapillary Atrophy in the Non-Glaucomatous and Non-Highly Myopic Participants of the Beijing Eye Study.

	Alpha Zone			Beta Zone		
Parameter	*P*-Value	Odds Ratio or Mean Difference	95% Confidence Interval of Odds Ratio or Diff.	*P*-Value	Odds Ratio or Mean Difference	95% Confidence Interval of Odds Ratio or Diff.
Systemic Parameters						
Age	<0.001	7.81	3.5, 12.1	<0.001	8.4	7.1, 9.7
Gender	0.63			0.05	0.75	0.56, 1.00
Rural/Urban Area of Habitation	0.09			<0.001	1.82	1.33, 2.48
Level of Education	0.12			0.61		
Smoking	0.24			0.21		
Alcohol Consumption	0.39			0.46		
Body Height	0.80			0.70		
Body Weight	0.15			0.43		
Body Mass Index	0.16			0.28		
Diastolic Blood Pressure	0.18			0.002	1.38	0.50, 2.25
Systolic Blood Pressure	0.38			0.02	1.92	0.30, 3.53
Mean Arterial Blood Pressure	0.76			0.58		
Arterial Hypertension	0.64			0.07	1.32	0.98, 1.77
Blood Concentration of: Cholesterol	0.52			0.51		
Glucose	0.61			0.86		
High-Density Lipoprotein	0.29			0.31		
Low-Density Lipoproteins	0.92			0.67		
Diabetes Mellitus	0.49			0.59		
Diabetic Retinopathy	0.52			0.74		
Intraocular Pressure	0.70			<0.001	1.2	0.5, 1.8
Ocular Perfusion Pressure	0.41			0.06		
Disc Hemorrhage	0.70			0.001	4.05	1.62, 10.2
Refractive Error	0.15			<0.001	0.78	0.46, 1.10
Axial Length	0.32			0.001	0.57	0.05, 1.09
Central Corneal Thickness	0.92			<0.001	10.7	5.5, 15.9
Anterior Chamber Depth	0.52			0.75		
Optic Disc Size	0.39			0.003	0.10	0.03, 0.18
Size of Alpha Zone	0.053	0.28	0.00, 0.55	<0.001	0.21	0.11, 0.32
Incidence of Alpha Zone	-------			<0.001	11.1	4.1, 30.3
Size of Beta Zone	<0.001	1.01	0.58, 1.44	<0.001	1.31	1.04, 1.60
Incidence of Beta Zone	<0.001	11.1	4.1, 30.3	-------		

**Table 2:** Logistic binary regression analysis for the associations with the incidence of beta zone of parapapillary atrophy during a 5-year follow-up of the Beijing Eye Study.

Parameter	*P*-Value	Regression Coefficient	Odds Ratio (OR)	95% Confidence Interval of the OR
Age	<0.001	0.11	1.12	1.10, 1.14
Intraocular Pressure	<0.001	0.09	1.10	1.05, 1.14
Refractive Error	<0.001	−0.34	0.71	0.67, 0.75
Rural/Urban Habitat	0.002	−0.54	0.58	0.41, 0.82
Central Corneal Thickness	0.02	0.006	1.01	1.00, 1.01
Presence of Glaucoma	<0.001	1.06	2.89	1.62, 5.14
Incidence of Alpha Zone	<0.001	1.96	7.13	2.43, 20.9
Arterial Hypertension	0.03	−0.36	0.70	0.51, 0.96
Gender	0.15	0.28	1.32	0.90, 1.94
Diastolic Blood Pressure	0.67	−0.009	0.99	0.95, 1.03
Systolic Blood Pressure	0.45	0.009	1.01	0.99, 1.03
Disc Hemorrhages	0.20	0.83	2.28	0.65, 8.07
Anterior Chamber Depth	0.32	0.32	1.38	0.74, 2.57

## Results

Of the 4439 subjects examined in 2001 and invited for re-examination in 2006, 3251 (73.2%) subjects returned for the follow-up examination, whereas 143 (3.2%) subjects were dead and 1045 (23.5%) subjects were alive but did not agree to be re-examined [25]. Of the study population of 2006 and taking into account the subject age in 2001, 1137 (35.0%) subjects were 40–49 years old, 896 (27.6%) were 50–59 years old, 923 (28.4%) were 60–69 years old, 276 (8.5%) were 70–79 years old, and 19 (0.6%) subjects were 80 years or older. Taking the population of China according to China's Inter-Census Survey in 2005 and considering only subjects with an age of 40+ age, 37.6% of the population (as compared 35.0% in our study) was in the age range of 40–49 years, 30.7% (as compared to 27.6%) was in the age range of 50–59 years, 17.7% (as compared to 28.4%) was in the age range of 60–69 years, 10.7% (as compared to 8.5%) was in the age range of 70–79 years, and 3.3% (as compared to 0.6%) was older than 80 years. In the youngest age group (40–49 years) of our study population, those subjects who resided in a rural setting constituted 63% of the study population, while in an older age group (60–69 years), the urban population was in the majority (71% versus 29%). Non-participants were significantly older (62.6±11.4 versus 60.4±10.1 (age in 2006); *P*<0.001) than were participants in the 2006 survey, lived predominantly in an urban region (64.9% versus 53.8%; *P*<0.001), and their mean best corrected visual acuity in the better-seeing eye was significantly worse (*P*<0.001). They did not vary significantly by gender (*P* = 0.49).

Assessable optic disc photographs taken at baseline and at the follow-up examination in 2006 were available for 3039 (93.5%) subjects. The overall rate of progression of alpha zone was 0.6±0.1% (95%CI: 0.3, 0.9) and of beta zone 8.2±0.5% (95%CI: 7.2, 9.1). A reduction in the size of alpha zone or beta zone was generally not observed. Since glaucoma is associated with an enlargement of parapapillary atrophy [3]–[21], we divided the whole study population into a glaucomatous subgroup (101 subjects) and a non-glaucomatous subgroup including the remaining subjects (n = 2938). The statistical analysis was performed separately in the two subgroups.

### Non-Glaucomatous Group

In the non-glaucomatous group, parapapillary atrophy did not differ between the baseline photography and the follow-up photography in 2705 (92.1%) subjects, while 9 (0.3%) subjects showed an enlargement of alpha zone only, 216 (7.4%) subjects showed an enlargement of beta zone only, and 8 (0.3%) subjects showed an enlargement of both alpha zone and beta zone. The progression rate of alpha zone was 0.6±0.1% (95%CI: 0.3, 0.9) and of beta zone 7.6±0.5% (95%CI: 6.7, 8.6). Since highly myopic eyes as compared with non-highly myopic eyes exhibit marked differences in their optic nerve head morphology including a large myopic crescent [29], the non-glaucomatous group was further subdivided into subjects with a myopic refractive error exceeding −8 diopters (n = 34 subjects) and the remaining subjects (n = 2904).

In the non-highly myopic non-glaucomatous subgroup, parapapillary atrophy did not differ between baseline and follow-up in 2695 (92.8%) subjects, while 9 (0.3%) subjects showed an enlargement of alpha zone only, 192 (6.6%) subjects showed an enlargement of beta zone only, and 8 (0.3%) subjects showed an enlargement of both alpha zone and beta zone. In this non-glaucomatous, non-highly myopic group, progression rate of alpha zone was 0.6±0.1% (95%CI: 0.3, 0.9) and of beta zone 6.9±0.5% (95%CI: 6.0, 7.8).

In univariate analysis, progression rate of alpha zone was significantly associated with increasing age (*P*<0.001), size of pre-existing beta zone (*P*<0.001), and co-progression of beta zone (*P*<0.001) (Table 1). Progression rate of beta zone was significantly associated with increasing age (*P*<0.001), rural area of habitation (*P*<0.001), lower diastolic blood pressure (*P* = 0.002), higher systolic blood pressure (*P* = 0.02), higher intraocular pressure (*P*<0.001), prevalence of disc hemorrhages (*P* = 0.001), more myopic refractive error (*P*<0.001), longer axial length (*P* = 0.001), higher corneal thickness (*P*<0.001), larger optic disc size (*P* = 0.003), larger area of alpha zone (*P*<0.001) and beta zone (*P*<0.001) at baseline, and co-progression of alpha zone (*P*<0.001) (Table 1). If a Bonferroni procedure was carried out to correct for performing multiple statistical comparisons and if thus the *P*-values were multiplied with the number of comparisons performed (n = 31), the relationships between progression rate of alpha zone and age, size of pre-existing beta zone, and co-progression of beta zone remained to be statistically significant. For the progression rate of beta zone, the relationships with age, rural area of habitation, higher intraocular pressure, prevalence of disc hemorrhages, more myopic refractive error, longer axial length, higher corneal thickness, larger area of alpha zone and beta zone at baseline, and co-progression of alpha zone remained to be statistically significant.

A binary regression analysis performed as second step of the statistical analysis, included the progression rate of alpha zone as dependent variable, and as independent variables all parameters, for which the *P*-value was less than 0.10 in the univariate analysis of their association with progressive alpha zone. It revealed that progressive alpha zone was still significantly associated with higher age (*P* = 0.02), co-progression of beta zone (*P* = 0.003), and size of beta zone at baseline (*P* = 0.05).

A similar binary regression analysis performed for the progression rate of beta zone revealed that progressive beta zone was significantly associated with higher age (*P*<0.001; OR: 1.09; 95%CI: 1.06, 1.12), rural region of habitation (*P* = 0.02; OR: 0.56; 95%CI: 0.35, 0.90), absence of arterial hypertension (*P* = 0.001; OR: 0.44; 95%CI: 0.27, 0.70), higher intraocular pressure (*P* = 0.001; OR: 1.11; 95%CI: 1.05, 1.19), higher myopic refractive error (*P* = 0.02; OR: 0.86; 95%CI: 0.77, 0.97), thicker central corneal thickness (*P* = 0.03; OR: 1.01; 95%CI: 1.00, 1.01), and larger pre-existing alpha zone (*P* = 0.001; OR: 1.47; 95%CI: 1.16, 1.85), and beta zone (*P*<0.001; OR: 2.15; 95%CI: 1.79, 2.57).

In the highly myopic non-glaucomatous subgroup, beta zone enlarged in 24 (70.6±7.9%; 95%CI: 54.4, 86.7) of the 34 subjects. The difference between highly myopic non-glaucomatous subgroup and the non-highly myopic non-glaucomatous subgroup in the progression rate of beta zone was significant (*P*<0.001; OR: 32.5; 95%CI: 15.3, 68.8). Due to the relatively small number of highly myopic eyes in the study population, a more detailed analysis of potential associations between progression of beta zone and other parameters was statistically not useful.

### Glaucomatous Group

In the glaucomatous group, parapapillary atrophy did not differ between the baseline photography and the follow-up photography in 77 (76.2%) subjects, while 23 (22.8%) subjects showed an enlargement of beta zone only, none of the subjects showed an enlargement of alpha zone only, and 1 (1.0%) subject showed an enlargement of both alpha zone and beta zone. In the glaucomatous group, parapapillary atrophy did not differ between the baseline photography and the follow-up photography in 77 (76.2%) subjects, while 23 (22.8%) subjects showed an enlargement of beta zone only, and 1 (1.0%) subject showed an enlargement of both alpha zone and beta zone. The progression rate of alpha zone was 1.0±1.0% (95%CI: 0.0, 3.0) and of beta zone 23.8±4.3% (95%CI: 15.3, 32.2). One of the subjects in the glaucomatous group was highly myopic, and this subject showed a progression of beta zone. The frequency of a progression of beta zone was significantly higher in the glaucomatous group than in the non-glaucomatous group before exclusion of highly myopic subjects (*P*<0.001; OR: 3.78; 95%CI: 2.34, 6.09) and after exclusion of highly myopic subjects (*P*<0.001; OR: 4.04; 95%CI: 2.48, 6.58). The progression rate of alpha zone did not differ significantly between the glaucomatous and the non-glaucomatous group (*P* = 0.46).

Including all study participants into the statistical analysis and performing a binary regression analysis revealed that progression rate of alpha zone was significantly associated higher age (*P* = 0.04) and the co-progression of zone beta (*P*<0.001). It was not significantly associated with gender (*P* = 0.57), intraocular pressure (*P* = 0.92), refractive error (*P* = 0.63), presence of glaucomatous optic nerve damage (*P* = 0.62), region of habitation (*P* = 0.26), central corneal thickness (*P* = 0.55), and area of alpha zone at baseline (*P* = 0.33). In a similar statistical analysis, frequency of progression of beta zone was significantly associated higher age, higher intraocular pressure, higher myopic refractive error, rural region of habitation, presence of glaucomatous optic nerve damage, absence of arterial hypertension, co-progression of alpha zone, and thicker central corneal thickness (Table 2). It was no longer significantly associated with gender, presence of optic disc hemorrhages, and anterior chamber depth (Table 2). Subjects with a non-glaucomatous optic nerve damage (n = 22) as compared to the remaining subjects did not vary in the progression rate of alpha zone (0.0% versus 0.6±0.1%; *P* = 1.0) and beta zone (8.2±0.5% versus 6.3±0.6%; *P* = 1.0).

## Discussion

In this population-based study of adult Chinese, we found that the overall 5-year progression rate of alpha zone was 0.6±0.1% and of beta zone 8.2±0.5%. The frequency of progression of alpha zone was significantly associated higher age and the co-progression of zone beta. It was not significantly associated with intraocular pressure, refractive error, and presence of glaucomatous optic nerve damage. The frequency of progression of beta zone was significantly correlated with higher age, rural region of habitation, absence of arterial hypertension, higher intraocular pressure, higher myopic refractive error, thicker central corneal thickness, and presence of glaucoma. It was not associated with non-glaucomatous optic nerve damage.

The results of our study on the progression of parapapillary atrophy and the associated factors agree with findings of previous cross-sectional studies on the prevalence of parapapillary atrophy in various population groups and with observations on factors associated with the presence of parapapillary atrophy. As shown in several previous hospital-based studies, beta zone was associated with glaucomatous optic neuropathy [3]–[21], [23]. As also reported in previous hospital-based longitudinal investigations of glaucoma patients, alpha zone was not related with the presence of glaucomatous optic neuropathy nor with intraocular pressure in our study. In previous longitudinal investigations, the development or enlargement of beta zone was timely and spatially associated with the progression of glaucoma. Patients with progressive glaucomatous optic nerve damage showed an enlargement of beta zone, often spatially correlated to the sector of the optic nerve head in which the neuroretinal was lost and corresponding to the location of the progressive visual field defect. To cite an example, Tezel and associates examined optic disc photographs of 305 ocular hypertension eyes with a follow-up of 10 years [9], [10]. An enlargement of parapapillary atrophy was detected in 25 (9.9%) of 252 non-progressive eyes and in 48 (49%) of 98 eyes developing open angle glaucoma. In a study performed by Uchida and co-workers, all 75 glaucoma eyes had parapapillary atrophy at baseline [11]. An enlargement of parapapillary atrophy was noted after a minimum follow-up of 4 years in 28 eyes. These included 21 (63%) of 33 eyes with signs of glaucomatous disc progression. The investigations mentioned above differed in the reported progression rate of parapapillary atrophy, with the potential reasons of differences in the study populations, definitions of parapapillary atrophy, and length of follow-up. Also in some studies, parapapillary atrophy was defined as sum of alpha zone and beta zone or only beta zone was assessed, while in our study, alpha zone and zone beta were assessed separately. None of the studies clarified the reasons for the temporal and spatial correlation between the prevalence and progression of parapapillary atrophy outside of the optic disc and the glaucomatous neuroretinal rim loss inside of the optic disc.

The present study also agrees with previous cross-sectional clinical studies and longitudinal experimental investigations that non-glaucomatous optic nerve damage is not associated with an increase in the size and frequency of alpha zone and beta zone of parapapillary atrophy [5], [30]. To mention an example, in monkeys with experimental temporary central retinal artery occlusion, Hayreh and colleagues did not observe an enlargement of parapapillary atrophy despite of marked non-glaucomatous optic nerve damage [31]. In a parallel manner, patients with non-glaucomatous optic nerve atrophy due to various reasons did not differ in frequency and size of alpha zone and beta zone of parapapillary atrophy from normal subjects [5]. It indicates that the development and enlargement of parapapillary atrophy is not associated with a loss of optic nerve fibers, but that glaucoma-related specific parameters may be responsible for the association between parapapillary atrophy and glaucoma.

After adjusting for parameters such as refractive error, presence of glaucoma, and area of habitation, the progression rate of both alpha zone and beta zone increased with higher age in the present study. It may be related to the physiologic age-related loss of about 0.3% of retinal photoreceptors and retinal pigment epithelium cells per year of life [32]. The finding may have clinical importance in that an enlargement of beta zone over time in patients with glaucoma may not necessarily indicate a progression of glaucoma but may also be explained by the increase in age.

The association between the progression of beta zone and myopic refractive error is paralleled by the general association of parapapillary atrophy with myopia [33]. In that aspect, it may be of importance that beta zone in a moderately myopic eye differs histologically from a beta zone in highly myopic eyes [34]. In non-highly myopic eyes, Bruch's membrane with underlying choroid extends to the edge of the optic disc, while in highly myopic eyes, the parapapillary region can be composed of a markedly thinned peripapillary scleral flange and retinal nerve fiber layer tissue, without Bruch's membrane and associated choroid [34]. These pronounced histological differences between a beta zone in non-highly myopic eyes and the beta zone in highly myopic eyes, despite similarities in the ophthalmoscopic appearance, indicate marked a difference in the pathogenesis. It may explain why the progression rate of beta zone was significantly higher in the highly myopic group.

The finding, that the progression rate of alpha zone and the progression rate of beta zone were correlated with each other, indicated that both zones were associated with each other. This is paralleled by clinical studies on patients with glaucoma, in which an alpha zone at the optic disc border was replaced by a new beta zone, while a (new) alpha zone had developed at the outer border of the newly formed beta zone [14]. It has remained unclear why the progression of beta zone was associated with rural region of habitation and with higher corneal thickness after adjustment for age, arterial hypertension, intraocular pressure, refractive error and presence of glaucoma.

Potential limitations of our study should be mentioned. First, the major concern, and a problem inherent to any epidemiologic study, is non-participation. The Beijing Eye Study 2006 had a reasonable response rate of overall 73%, 3251 of the 4439 subjects participated in the follow-up survey (or 61% of the group originally eligible in 2001); however, differences between participants and non-participants could have led to a selection artifact, and may have produced bias in the progression rates of alpha zone and beta zone of parapapillary atrophy. Second, 90.9% of the study population was younger than 70 years (40–69), 8.5% of the study population was 70–79 years old, and only 0.6% (19 persons) was 80 years and older at baseline. As compared with the total Chinese population, our study population had a relatively overweight in the age group of 60–69 years (28.4% in our study population versus 17.7% in the total Chinese population), and a relative underweight in the age group of 80+ years (0.6% versus 3.3%). The relatively low participation among the older, especially those older than 80 years, may have led to an underestimation of the progression rate of parapapillary atrophy. Third, another limitation of the present study may be the question how representative the rural regions and the urban regions of Greater Beijing are for the entire area of China. Potential strengths of our study may be first that it is a population-based study with a relatively large sample size and a high participation rate (73%). Second, our study is the first investigations to look at progression rates of parapapillary atrophy worldwide.

In conclusion, in adult Chinese in Greater Beijing, the 5-year frequency of an enlargement of beta zone of parapapillary atrophy (8.2±0.5%) was significantly correlated with higher age, rural region of habitation, absence of arterial hypertension, higher intraocular pressure, higher myopic refractive error, thicker central corneal thickness, and presence of glaucoma. It was not associated with non-glaucomatous optic nerve damage.
